# Understanding health care pathways of patients with sepsis: protocol of a mixed-methods analysis of health care utilization, experiences, and needs of patients with and after sepsis

**DOI:** 10.1186/s12913-023-10509-4

**Published:** 2024-01-08

**Authors:** Carolin Fleischmann-Struzek, Norman Rose, Bianka Ditscheid, Lea Draeger, Patrik Dröge, Antje Freytag, Ludwig Goldhahn, Lena Kannengießer, Aurelia Kimmig, Claudia Matthäus-Krämer, Thomas Ruhnke, Konrad Reinhart, Peter Schlattmann, Konrad Schmidt, Josephine Storch, Ruben Ulbrich, Susanne Ullmann, Lisa Wedekind, Enno Swart

**Affiliations:** 1 Institute of Infectious Diseases and Infection Control, Jena University Hospital, Stoystraße 3, 07743 Jena, Germany; 2 Center for Sepsis Control and Care, Jena University Hospital, Jena, Germany; 3 Institute of General Practice and Family Medicine, Jena University Hospital, Jena, Germany; 4 AOK Research Institute (WIdO), Berlin, Germany; 5 Institute of Social Medicine and Health Systems Research (ISMHSR), Otto-Von-Guericke-University Magdeburg, Magdeburg, Germany; 6 Sepsis Foundation, Berlin, Germany; 7 Department of Anesthesiology and Operative Intensive Care Medicine, Charité University Medicine Berlin, Berlin, Germany; 8 Institute of Medical Statistics, Computer and Data Sciences, Jena University Hospital, Jena, Germany; 9 Institute of General Practice and Family Medicine, Charité University Medicine Berlin, Berlin, Germany

**Keywords:** Sepsis, Septic shock, Health service research, Qualitative research, Mixed-methods, Barriers, Early recognition, Interview, Emergency medical services, Aftercare, Post-Sepsis Syndrome

## Abstract

**Background:**

Sepsis is associated with about 20% of deaths worldwide. It often presents with non-specific initial symptoms, making its emergency treatment an interdisciplinary and cross-sectoral challenge. Three in four sepsis survivors suffers from new cognitive, psychological, or physical sequelae for which specific treatment concepts are scarce. The AVENIR project aims to improve the understanding of patient pathways, and subjective care experiences and needs along the entire healthcare pathway before, with and after sepsis. Based on this, concrete recommendations for the organization of care and patient information materials will be developed with close patient participation.

**Methods:**

Mixed-methods study including (1) analysis of anonymized nationwide health claims data from Germany, (2) linkage of health claims data with patient care reports (PCR) of emergency medical services from study regions in two federal states within Germany, and (3) qualitative exploration of the patient, relative, and care provider perspective on sepsis care. In (1), we analyze inpatient and outpatient health care utilization until 30 days pre-sepsis; clinical sepsis care including intra- and inter-hospital transfers; and rehabilitation, inpatient and outpatient aftercare of sepsis survivors as well as costs for health care utilization until 24 months post-sepsis. We attempt to identify survivor classes with similar health care utilization by Latent Class Analyses. In (2), PCR are linked with health claims data to establish a comprehensive database outlining care pathways for sepsis patients from pre-hospital to follow-up. We investigate e.g., whether correct initial assessment is associated with acute (e.g., same-day lethality) and long-term (e.g., new need for care, long-term mortality) outcomes of patients. We compare the performance of sepsis-specific screening tools such as qSOFA, NEWS-2 or PRESEP in the pre-clinical setting. In (3), semi-structured interviews as well as synchronous and asynchronous online focus groups are conducted and analyzed using qualitative content analyses techniques.

**Discussion:**

The results of the AVENIR study will contribute to a deeper understanding of sepsis care pathways in Germany. They may serve as a base for improvements and innovations in sepsis care, that in the long-term can contribute to reduce the personal, medical, and societal burden of sepsis and its sepsis sequelae.

**Trial registration:**

Registered at German Clinical Trial Register (ID: DRKS00031302, date of registration: 5th May 2023).

## Background

Sepsis is the most serious complication of infectious diseases, in which organ failure and shock occur due to a dysregulated immune response of the body to any bacterial, viral, fungal or parasitic infection [1]. Annually, an estimated 49 million patients develop sepsis globally, of which 11 million die [2]. In Germany, approximately 150,000 patients were hospitalized with sepsis in 2016; the in-hospital case fatality was 40% [3]. In the long-term, three out of four sepsis survivors suffer from new-onset physical, psychological, and/or cognitive sequelae in the year post-sepsis. These symptoms are summarized as Post-Sepsis Syndrome (PSS) [4] and include neuromuscular, cardiovascular, and urogenital disorders as well as depression, fatigue, and cognitive impairment [5]. Given the large number of possible (severe) sequelae, a high proportion of sepsis survivors is rehospitalized after recovering from the initial septic insult, often for re-emerging infections, sepsis and cardiovascular disease [6, 7]. Of these rehospitalizations, 22% occurred with so-called ambulatory-care sensitive diagnoses according to an US-study [6], many of which are considered potentially preventable, for example, through vaccination [6]. The costs incurred by acute treatment and long-term care of those suffering from sepsis annually in Germany up to three years after the index illness amount to approximately 12 billion Euro, making sepsis acute and long-term care a medical and societal challenge [4].

To a high-quality acute and long-term sepsis care, however, there are numerous challenges. First, given its non-specific early symptoms, sepsis is often recognized and treated with delay, [8] resulting in a considerable number of preventable deaths. Second, a multitude of disciplines are involved in pre-clinical, acute, and post-acute sepsis care, which increases the risk of delays and loss of information [9]. Third, for the large number of sepsis survivors with sequelae, there is a lack of widely available and effective aftercare options that can address the diverse spectrum of physical, cognitive, and psychological sequelae [10]. This may be one of the reasons why only a small proportion of sepsis patients (17%) receive rehabilitation after their acute care hospitalization in Germany [11]. A survey among sepsis survivors also revealed that such rehabilitation often addresses only a small proportion of the sequelae [12]. Specific rehabilitation concepts for sepsis survivors are lacking, and survivors call for better and more specific follow-up care [13].

In view of these deficiencies in care, the WHO called on its member states in 2017 to improve the prevention, diagnosis, and adequate treatment of sepsis and its consequences [14]. A prerequisite for the development and implementation of effective care strategies, however, is the improved understanding of care pathways and experiences of sepsis patients, which have been insufficiently studied to date. The AVENIR project therefore aims to (i) analyze care pathways of patients with and after sepsis in the German health care system, (ii) identify patient classes with similar patterns of health care utilization after sepsis and examine their association with sepsis, patient, and treatment characteristics, and (iii) describe subjective care experiences and needs of sepsis patients and health care providers. Based on this evidence, materials for patient information will be developed and concrete recommendations for the organization of health care will be derived.

## Methods/design

**Fig. 1:**
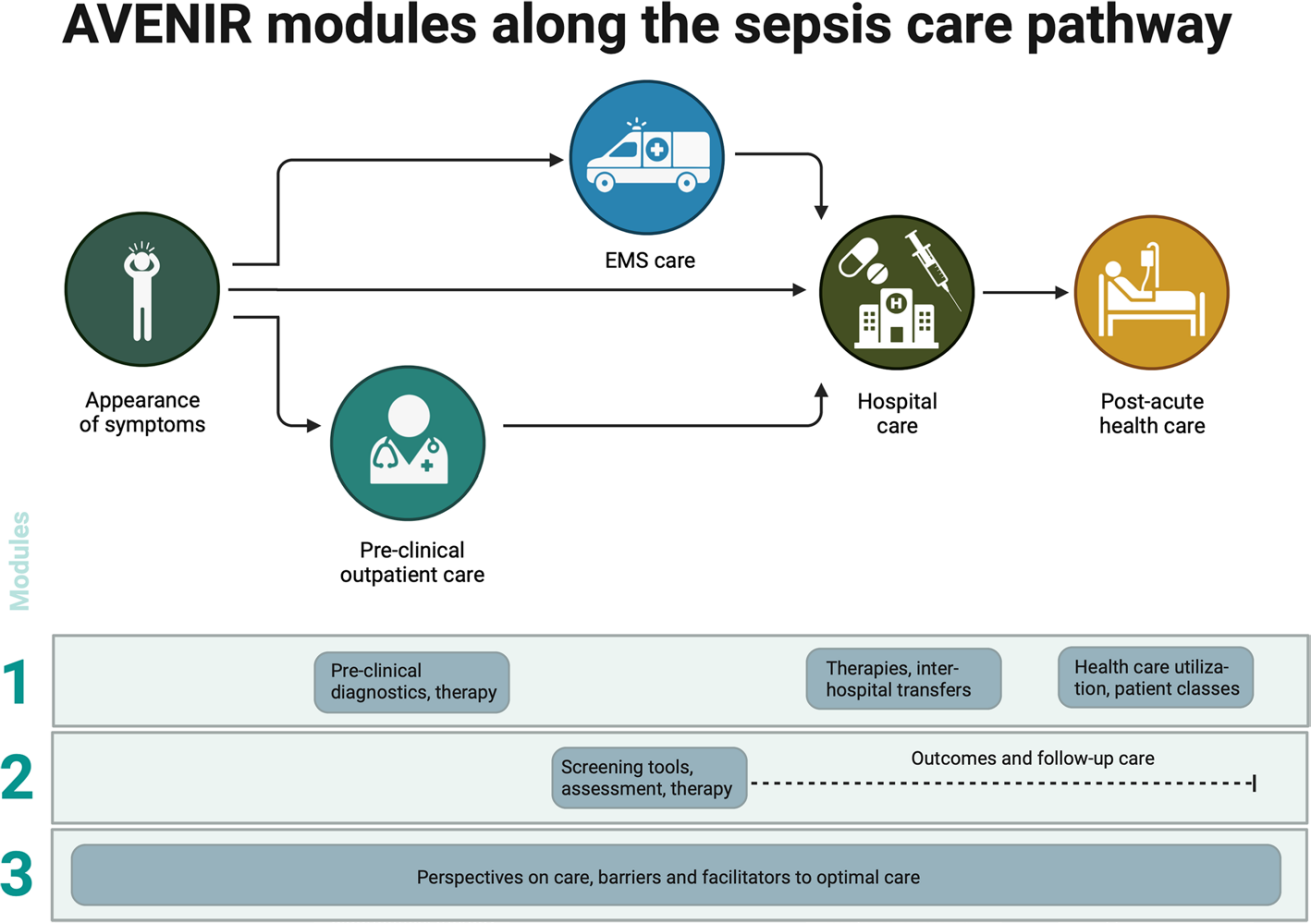
AVENIR modules by sepsis care pathway

AVENIR is a mixed-method, non-interventional study, which consists of three modules (Fig. 1). The study was pre-registered in the German Clinical Trials Registry (ID: DRKS00031302).

### Module 1: Pre-clinical, acute and post-acute health care pathways—analysis of anonymized nationwide health claims data

#### Design and data source

In module 1, we perform a population-based observational cohort study using health claims data from eleven legally independent statutory health insurance funds of the ”AOK – Die Gesundheitskasse” (local health care funds) covering around one third of the German population. Enrolment into statutory health insurance is unrestricted regarding age, health status, income, employment, or geographical region. The routine data from 2015–2021 are provided by AOK Research Institute (WIdO), including a de-identified patient ID that allows for longitudinal analyses and linkage of data within the database. Administrative data for inpatient and outpatient care (§ 301 Social Code Book V (SGB V), §295 SGB V) as well as for rehabilitation therapies (§ 301 SGB V), outpatient medication (§ 300 SGB V), medical remedies (§ 302 SGB V), sickness benefits payments (§295 SGB V), care in disease management programs (§ 137f SGB V), home nursing care (§ 302 SGB V) and benefits of the statutory long-term care insurance according to SGB V and XI are analyzed.

#### Patient sample

Patients > 15 years of age with hospital-treated sepsis in 2016–2020 are included, as identified by the following strategies based on primary or secondary ICD-10-GM hospital discharge diagnoses (Table 1).

**Table 1:** Sepsis definition criteria in health claims data

Year(s)	Sepsis and Infection ICD-10-GM Codes	Organ dysfunction ICD-10-GM or OPS Codes
2016–2019	R57.2 (septic shock) OR	
	R65.1 (severe sepsis) OR	
	At least one explicit sepsis code (A02.1, A20.0, A20.7, A21.7, A22.7, A24.1, A26.7, A28.2, A32.7, A39.1, A39.2, A39.3, A39.4, A40, A41, A42.7, A48.3, A49.9, A54.8, B00.7, B37.6, B37.7, B49, O75.3, O85, T80.2, T81.4, T88.0)	AND at least one ICD-10-GM code for organ dysfunction (J80.0, J95.1, J95.2, J96.0, J96.9, R09.2, D65, D69.57, D69.58, D69.59, D69.6, R57.8, E86, I95.9, I46.0, I46.9, R57.2, K72.0, K72.9, R17, N17.02, N17.03, N17.12, N17.13, N17.22, N17.23, N17.82, N17.83, N17.92, N17.93, F05, G94.32, K72.72!, K72.73!, K72.74!, R40, G93.4) OR OPS code for organ dysfunction (8–701,^a^ 8–704,^a^ 8–706,^a^ 5–311,^a^ 5–312,^a^ 8–800.6, 8–800.d, 8–800.f, 8–800.g, 8–800.h, 8–800.j, 8–800.k, 8–800.m, 8–800.n, 8–810.j, 8–810.x, 8 812.5, 8–812.6, 8–812.8, 8–771, 8–779, 8–852, 8–858, 8–821.2, 8–853.1, 8–853.3, 8–853.4, 8–853.5, 8–853.6, 8–853.7, 8–853.8, 8–853.x, 8–853.y, 8–854.2, 8–854.3, 8–854.4, 8–854.5, 8–854.6, 8–854.7, 8–854.8, 8–854.x, 8–854.y, 8–855.1, 8–855.3, 8–855.4, 8–855.5, 8–855.6, 8–855.7, 8–855.8, 8–855.x, 8–855.y, 8–856, 8-85a.0, 8-85a.1)
2020	R57.2 (septic shock) OR	
	At least one explicit sepsis code (see above)	AND R65.1 (SIRS with organ dysfunction) OR
	At least one explicit sepsis code (see above)	AND at least one ICD-10-GM code for organ dysfunction (see above) OR OPS code for organ dysfunction (see above) OR
	J09 OR J10 (laboratory confirmed Influenza) OR U07.1! (laboratory confirmed SARS-CoV2-infection)	AND at least one ICD-10-GM OR OPS code for organ dysfunction (see above)

*Abbreviations*: *ICD-10* International Classification of Diseases Version 10, German Modification, *OPS* German Procedure Classification, Operationen- und Prozedurenschlüssel

^a^codes were included in the definition if at least one hour of mechanical ventilation was documented in the course of the hospitalization

ICD-10-GM for sepsis codes were consistently defined by sepsis-1 definition [15] until the end of 2019. In 2020, sepsis coding was adjusted to the new sepsis-3 definitions [1]. To address these changes and to capture patients with viral sepsis, for which no explicit sepsis code is available since the change of the definitions, we extended our case identification strategies to cases with a code for Covid-19 or Influenza and a code indicating an acute organ dysfunction. The first hospital case of a patient with sepsis in the observation period is referred to as the index treatment; possible following sepsis cases are considered as recurrent sepsis. Only patients with continuous insured status 12 months before and 24 months (12 months for the 2020 patient cohort) after index treatment with sepsis or until death are included in the study.

#### Sample size considerations

Based on preliminary studies using the same database and similar inclusion criteria, [4] we expect approximately 80,000 sepsis patients per year between 2016–2019. During the pandemic, the number of Non-Covid-19 sepsis patients may be lower, [16] but due to the inclusion of Covid-19 patients with organ dysfunction, we expect a similar number of patients to be included in our cohort in 2020. Patients with AOK insurance can receive treatment in any acute care hospital in Germany. According to a preliminary analysis, AOK-insured sepsis patients were treated in around 1650 hospitals of the 1900 German acute care hospitals between 2016–2020, thus our study will cover a large majority of German hospitals.

#### Measurements

The module focuses on the three main points of the treatment pathway for sepsis patients: (A) pre-hospital care, (B) inpatient care, and (C) post-acute care.

(A) Pre-hospital care: Pre-hospital health care utilization of patients with community-acquired sepsis in the 30 and 7 days prior to hospital admission is assessed. Specifically, contacts with the outpatient care system are analyzed, for example, how often the general practitioner, any outpatient specialist or the statutory health insurance emergency service were consulted, what laboratory or Point of Care Testing (POCT) diagnostics were performed, and if any initial anti-infective therapy was prescribed. For these outpatient contacts, the time latency between the last contact and the following inpatient admission with sepsis is assessed. Furthermore, we analyze which proportion of sepsis patients had pre-sepsis hospitalizations or emergency department visits, as well as which proportion of sepsis patients was admitted as emergency to their index treatment, and for which admission diagnosis.
(B) Inpatient care: For the index hospitalization, we assess treatment characteristics, e.g. which organ replacement therapies were conducted, if patients received any immune absorbent therapy, if an intensive care complex or palliative care treatment was administered; as well as the departments involved in sepsis care. We identify inter-hospital transfers and characterize hospitals involved in the chain of sepsis care by the following key characteristics: type of hospital ownership (owned by local or federal state authorities, private for-profit organizations, or by voluntary charitable organizations or churches) number of hospital cases and beds, region (urban/rural), number of cases with (complex) intensive care complex treatment.
(C) Post-acute care: Post-acute care utilization is assessed starting at the day of discharge from the last interhospital-transfer from the index hospitalization. We analyze long-term mortality, rehospitalizations (in general, for infectious diseases or recurring sepsis, for vaccine-preventable diseases, for ambulatory-care sensitive diagnosis), emergency department visits in hospitals or treatment by emergency services of the statutory health insurance emergency service, inpatient rehabilitation therapies, utilization of medical remedies, as well as outpatient consultations. We put special emphasis on the out-of-hospital intensive care and the conduction of long-term mechanical ventilation in that setting. Furthermore, psychotherapy and psychiatric medications are recorded and parameters concerning the return to work. Based on a detailed description of this post-acute sepsis care, we attempt to identify classes of health care utilization among sepsis survivors, examine whether such classes are constant or variable over time, and whether specific patient, sepsis, and treatment characteristics are associated with class membership.

For (A), (B) and (C), we examine health care costs from a payer perspective. All analyses will be conducted for the complete cohort of sepsis patients as well as for relevant subgroups, e.g. for patients stratified by age, sepsis focus, underlying pathogen (Covid-19 vs. Influenza vs. others), severity, region, underlying comorbidity, or patients with/without new impairments post-sepsis.

#### Statistical analyses

We present descriptive analyses to describe the extent of health care utilization of different health services after sepsis and use visualization methods to map health care pathways among survivors, such as Sankey diagrams. Sankey diagrams are used to visualize patients’ pathways in terms of provider contacts after the sepsis event. This presentation allows us to detect patient flows after a hospital visit and provides support to define more detailed research questions [17]. Associations between hospital characteristics involved in the chain of hospital care and acute and long-term outcomes, as well as between treatment characteristics and the avoidance of (ambulatory-care sensitive) rehospitalizations are assessed by using appropriate regression models.

We attempt to describe the heterogeneity in health care utilization among sepsis survivors by Latent Class Analysis (LCA) modelling, which has proven as a promising tool to identify subgroups of patients with different patterns of health service use in previous research [18]. We will assess the stability of the class analysis over the follow-up period and study potential change in latent class membership by latent transition analysis (LTA) [19]. Additional analyses aim at explaining the heterogeneity in health care utilization by individual characteristics of the patients (e.g., age, sex, co-morbidities) and the sepsis (e.g., organ dysfunctions, duration of mechanical ventilation) by including these variables as predictors of the latent class membership in a multinomial logistic regression. Furthermore, the latent class membership may predict short and long-term outcomes, which can also be analyzed using regression based approaches with the latent classes as predictor of distal outcomes as described by Bakk and Kuha [20].

### Module 2: Patient care reports of emergency medical services and health claims data – separate and linked analysis

#### Design and data source

Module 2 is a population-based cohort study formed by patients who are cared for by emergency medical services (EMS) in the two German federal states Bayern (BY) and Baden-Württemberg (BW). In both states, standardized documentation in EMS as well as extensive efforts for quality assurance have existed for quite some time [21].

As first data source, patient care reports (PCR) of rescue drives conducted by paramedics and/or emergency physicians will be acquired from four ground-based EMS organizations. Activities from 29 EMS districts are incorporated. In addition, PCR of one supra-regional air-based EMS organization will be included. In Germany, paramedics can carry out rescue operations independently or in collaboration with emergency physicians. The latter either accompany the operation right from the start or are only called upon if a need arises during the deployment. On the local level, several EMS organizations may operate simultaneously.

**Fig. 2:**
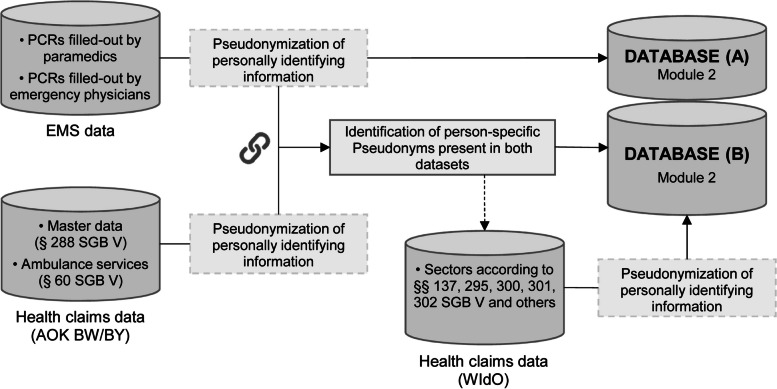
Data sources, linkage and resulting databases in module 2 of AVENIR. Abbreviations: AOK: Statutory Health Insurance Fund, AOK – Die Gesundheitskasse; BY: Bayern, Federal State, BW: Baden-Württemberg, Federal State, EMS: Emergency Medical Services, PCR: Patient Care Report, SGB: Social Code Book, Sozialgesetzbuch, WIdO: AOK Research Institute, Wissenschaftliches Institut der AOK

As second data source, the statutory health insurance funds AOK BW and AOK BY which have the largest market share in the respective federal states with more than 4 million insured persons each, provide health claims data on the person level. The PCR are linked to health claims data via health insurance number (KVNR) pseudonymized with the widely used SHA-256 algorithm. In case the pseudonymized KVNR of a person is missing, linkage is conducted using indirect key variables following an approach developed by Goldhahn et al. [22] For insured persons for whom a successful linkage is possible, additional health claims data will subsequently be provided by AOK Research Institute WIdO as third data source. The health claims data delivered by WIdO are consistent with the sections of SGB V and XI listed in Module 1. The initial data sources, the linkage process as well as the resulting databases are depicted in Fig. 2.

Module 2 is based on (A) analyses of the PCR and (B) analyses on the linked data (PCR and health-claims data), which allows to trace care pathways and outcomes of sepsis patients from the pre-hospital setting onwards in short as well as long-term.

#### Patient sample

(A) The patient sample includes all EMS cases treated by cooperating EMS organizations in Bayern and Baden-Württemberg between January 1, 2017, and December 31, 2021.

(B) The patient sample consists of individuals for whom a linkage can be established between PCR and health claims data. The health claims data cover the period from January 1, 2016, to December 31, 2022, thereby allowing for a one-year pre- or post-analysis of all patients' EMS-cases. The sample only includes data from individuals with a continuous insurance period or death within observation period.

In module 2, neither PCR nor health claims data are subject to sepsis-specific pre-selection. Consequently, the PCR comprise cases of patients with and without suspected sepsis, and the health claims database consists of patients with and without diagnosed sepsis.

#### Sample size considerations

Due to the varying characteristics of the study regions and EMS- organizations, it is challenging to forecast the number of expected cases. By extrapolating the experience from a research project comparable to AVENIR, [22] we expect approximately 2.8 million PCRs for module 2 (A). Around 40% of the population in the federal states of BY and BW is insured with the AOK. Thus, we estimate approximately 1.1 million cases can be linked to the health claims data for further analyses in (B). Consequently, given a sepsis rate of 2—4% in EMS, [23, 24] around 22.700—45.400 cases of patients with sepsis are to be expected.

#### Measurements

(A) The PCR are first examined with regard to their documentation quality. This includes, for example, the frequency of missing values, invalid values or non-plausible values. Checks of vital signs are especially important in this process, since they are often not documented even in case of suspected infection or sepsis [25].

We will investigate the proportion of patients with a documented suspicion of sepsis in the pre-hospital setting and the symptoms of these patients. We analyze what proportion of patients with suspected sepsis already receive care by emergency physicians in the pre-hospital setting, and how the pre-hospital care of sepsis patients with and without the involvement of emergency physicians differs. In addition, we investigate which therapies are initiated in the pre-hospital setting if sepsis is suspected (e.g., drug administration, oxygen administration, ventilation, administration of antibiotics, fluid therapy, catecholamines, vasopressors).

(B) Based on the linked data, we assess in which sectors (e.g. outpatient treatment, inpatient admission to a hospital) of the health care system patients received follow-up care after EMS treatment, and if this care differs with respect to sepsis suspicion during EMS treatment. Further analysis will determine whether sepsis suspicion in the pre-hospital setting affects short- and long-term patient outcomes (e.g., ventilation, mortality, readmission due to specific diagnoses, need for new care).

In addition, sepsis-specific screening tools such as qSOFA and others [26, 27] will be operationalized based on available vital signs in PCR. The aim is to evaluate how precise the pre-clinical screening tools detect sepsis and if this precision changed over time compared to a clinically confirmed and coded sepsis diagnosis (defined in accordance to Module 1) during a subsequent hospitalization.

A special focus lies on false-negative classifications, i.e. patients without a pre-clinical suspicion of sepsis but confirmed sepsis diagnosis in health claims data. For this group, potential sepsis characteristics (e.g. organ dysfunction by qSOFA) or patient characteristics will be investigated in more detail.

#### Statistical analyses

Data quality in the PCR is primarily examined with an exploratory approach. We plan to use methods for the assessment of data quality (e.g. visualization through heat maps). For analyses in (A) and (B) we use frequency counts, cross-tabulations, measures of location and dispersion measures.

The characteristics of pre-clinical sepsis screening tools are analyzed by using binary classifiers. Measures such as sensitivity, specificity, negative/positive predictive value, are calculated.

Additionally, descriptive measures and appropriate visualization techniques, such as Sankey plots, are utilized to depict pre-hospital patient pathways, including emergency care, inpatient admission or utilization of other health care sectors. This is performed for the total patient sample, but also for subgroups (e.g., by age, gender, comorbidity). In order to evaluate acute and long-term patient outcomes, module 2 employs descriptive and multivariate analysis methods, which are suitable for the data structure. Survival times of patients are investigated by using the Kaplan–Meier estimator.

### Module 3: Qualitative exploration of the patient and provider perspective on sepsis care

#### Design and data source

Module 3 consists of a qualitative approach focusing on (A) patients’ and their relatives’ as well as on (B) health care providers’ perspective on sepsis care. In 3 (A), semi-structured telephone interviews with patients and/or their relatives are planned to assess their individual perspective on pre-clinical, clinical, and post-acute care as well as their satisfaction with information and informational needs along this pathway. In 3 (B), health care professionals’ perspective is investigated using interdisciplinary focus group discussions, with a main focus on transsectoral transitions between pre-clinical, clinical, and post-acute care. For each transition (pre-clinical – clinical; clinical – post-acute care), 4 to 5 focus groups are planned. To inform the development of focus group guidelines, 9 upstream video-supported interviews with individuals of different health care professions involved in pre-clinical, clinical, and post-acute sepsis care are conducted.

#### Sample

Three (A) interview participants are recruited using advertising posts on social media platforms and flyer placement in the post intensive care unit of the Charité University Medicine Berlin. Patients >  = 18 years, who are fluent in German and who developed sepsis before a maximum of five years are eligible for participation. Patients are recruited until data saturation is being reached in the interviews. (B) Upstream interviews are planned with 9 health care professionals from different disciplines (pre-clinical: 3, clinical: 3, post-acute: 3). For the focus group discussion rounds, we aim to involve 6–8 participants per group, adding up to a maximum amount of 80 health care professionals in total. Each focus group will be multidisciplinary and cross-sectoral in the selection of participants. Participants for interviews and focus groups are identified by in-house databases as well as the website information of eligible institutions, and are contacted directly by phone or e-mail with an information flyer and additional details. Participation requires a professional experience of at least one year in the respective field. The participants receive an expense allowance of 60 Euro for the expert interviews and 120 Euro for the focus group discussions.

#### Measurement

(A) In line with our explorative, qualitative approach we intend to use open-ended questions. Thematically, the interview guides will be grounded on a systematic review carried out in advance as well as internal experts rounds and comprise topics like: Challenges, best practice examples, and deficits existing in acute and long-term care after sepsis, especially in the transitions between sectors. For details on the systematic review, we refer to the PROSPERO registration (CRD42023405681). (B) In upstream interviews, practices and subjective perceptions of sepsis care regarding four main thematic blocks (recognition, acute treatment, rehabilitation/aftercare, and transition of care, selected depending on the field of work of the interview participant) will be investigated using open-ended questions. An emphasis will be placed on perceived barriers and contributory factors to prompt and adequate care. Especially, desires for optimal care will be elaborated on. The interview guide development bases on a semi-systematic review of current literature. The drafted guide is discussed and revised within the project team as well as with a team of researchers and clinicians of different specializations at the Jena University Hospital, and pretested in a pilot interview. All upstream interviews will be audio-recorded, transcribed, and proofread.

Focus group discussions are centered on intersectoral transitions of care, particularly on multidisciplinary communication and collaboration among involved health care professionals, and aim to identify facilitators and barriers of successful intersector-transitions. In the course of the pandemic, new remote research approaches had to be established in which people interact without face-to-face contact [28]. This includes the development of asynchronous online focus groups (AOFG), which is a dynamic digital bulletin board method that takes place over a set number of days, e.g. 3 – 30 days [29] and where study moderators post different discussion prompts, and participants are able to respond at any time until the focus group closes [30]. In order to compare and evaluate synchronous and asynchronous online focus groups (SOFGs and AOFGs, respectively), we decided to use both approaches: AOFG for the transition pre-clinical to clinical care, and SOFG for the transition clinical to post-acute care [30]. Consequently, two separate but similar focus group guides are developed based on the upstream interviews and literature review. Each focus group guide will be pretested once. Due to the text format of AOFGs, transcription is not required. We use the Moodle platform of the Jena University Hospital for the AOFGs, from which data can be directly imported to MAXQDA. SOFGs are video-recorded, transcribed, and proofread. Both the interviews and focus groups are conducted by researchers experienced in qualitative research methods.

#### Analyses

Interviews in (A) are analyzed through the qualitative analysis approach according to Kuckartz [31]. In (B), the interviews are analyzed through the qualitative analysis approach according to Mayring [32]. Mayring's model is more theory-driven and thus more suitable for analyzing the upstream interviews, which are intended to help conceptualize the content of the focus groups, from our perspective. SOFGs are analyzed in two steps. First, aspects and experiences of the participants on a specific input are collected, discussed, and defined on moderation cards. Subsequently, the "codes" are categorized according to the principles of the Concept Mapping Approach [33–35] during the meeting. In this way, interpretation errors can be reduced. The discussions are video-recorded and transcribed. In the second step, the final transcripts will be analyzed using MAXQDA according to the framework approach [36] by two independent researchers. AOFGs are analyzed as described in the second step.

### Project timeline

The AVENIR project started in August 2022. To date (November 2023), the preparation phase is completed. This phase included the definition of inclusion criteria, and variables used, as well as the preparation of analysis plans in module 1 and 2. In module 3, literature reviews and upstream interviews informing the interview guidelines and pilot interviews were conducted and recruitment of participants has started. For all three modules, ethics and data protection votes were obtained. We plan for completing data collection and analyses by February 2025, and derivation of patient information and recommendations to improve sepsis care by August 2025.

## Discussion

Given the large number of sepsis patients and their families who have experience mortality and substantial long-term morbidity from sepsis, the improvement of sepsis care is considered a global public health priority [14]. The overarching aim of the AVENIR study is to advance the understanding of current sepsis care practices and patient needs by triangulation of the quantitative analysis of nationwide health claims data and EMS data, with the qualitative perspective from patient, caregiver and health care provider interviews and focus groups. By that, the AVENIR study generates nuanced knowledge on every phase of the care pathway from sepsis onset to long-term care. It will help to understand patients’ perception of optimal care, and providers perceptions of facilitators and challenges of delivering care in this way, particularly at intersectoral transitions. Furthermore, the study helps to identify patient-, sepsis- or treatment related factors during an acute stay associated with adverse outcomes or specific health care utilizations patterns, which may help to design and tailor interventions to support patients beyond their immediate discharge from hospital based on their specific needs. At a health system and policy level, the results may support resource allocation, capacity planning and the implementation of policies that enhance acute and longitudinal sepsis care.

A major strength of the study is its mixed-methods design and the comprehensive database including both primary and secondary data sources. We use health claims data from approximately one third of the German﻿ population, that records health care utilization in most German health care sectors (inpatient, outpatient, rehabilitation, medical remedies, chronic care). The data linkage of PCR of EMS and health claims data completes the database with data from two federal states and enables the follow-up of short- and long-term outcomes of EMS patients with and without sepsis. This also allows to explore the extent of unrecognized sepsis in EMS care and its impact on sepsis treatment and outcomes. Beyond the information drawn from health claims data, our study generates an immersion into the perspectives and needs of patients, family members, and providers.

Our study has also limitations to consider. On the one hand, these arise from inherent limitations of research using health claims data, such as potentially incomplete, inaccurate, or missing data, and inability to adequately evaluate the appropriateness of care [37, 38]. Although health care utilization and costs can be researched with high validity, recorded incidences of diseases have to be considered as administrative as only diseases diagnosed and coded correctly during hospitalizations and outpatients visits can be detected. For sepsis, we know from previous validation studies that the coding of diagnoses captures cases only with low sensitivity, and misses cases with lower disease severity [39]. This fact may be aggravated by the change in sepsis-related ICD-10-GM codes in 2020 in Germany. We address this issue by applying different case identification strategies for the years 2016–19 and 2020 (Table 1), but still it is likely that our sepsis definition does not capture all sepsis patients treated in German hospitals.

In module 2, exclusively PCR of EMS from study regions in Bayern and Baden-Württemberg as well as from selected EMS organizations will be processed. Hence, all generalizations of study results with regard to these two federal states or to the entire health care system in Germany must be examined carefully.

Furthermore, undesired selection effects may be introduced into the database in case key variables needed for the linkage of PCR and health claims data are unavailable selectively (e.g. KVNR). However, it is known from previous studies [22] that data linkage with health claims data is possible even if the key variable KVNR is not available for some of the PCR.

Qualitative data curation in module 3 might be limited by the fact that since participants of the oral interviews are not sharing statements anonymously in interviews and SOFGs, statements might not be free from social desirability effects. The sample might be subject to self-selection bias as participation will be voluntarily. Moreover, interviews using a video conference system may not reach the same depth as face-to-face interviews as rapport building can be limited. However, using online video-supported interviews allow for a composition of groups without logistic concerns and geographical restrictions, which increases the groups ‘ diversity and quality.

In conclusion, the results of the mixed-method study AVENIR contribute to a deeper understanding of sepsis care pathways in Germany. In the long-term, this may help to foster improvements and innovations in sepsis care, which can contribute to reduce the immense personal, medical, and societal burden of sepsis and its sepsis sequelae.

## Data Availability

Not applicable to the study protocol presented in this manuscript.

## Abbreviations

AOFG: Asynchronous online focus group
AOK: Statutory health insurance fund, AOK – Die Gesundheitskasse
BW: Baden-Württemberg, federal State
BY: Bayern, Federal State
EMS: Emergency medical services
ICD: International classification of diseases
KVNR: Health insurence number, Krankenversichertennummer
LCA: Latent class analysis
LTA: Latent transition analysis
OPS: German procedure classification, operationen- und prozedurenschlüssel
PCR: Patient care report
PSS: Post sepsis syndrome
SGB: Social code book, sozialgesetzbuch
SOFG: Synchronous online focus group
WIdO: AOK research Institute, Wissenschaftliches Institut der AOK

## References

[1] Singer M, Deutschman CS, Seymour CW (2016). The Third International Consensus Definitions for Sepsis and Septic Shock (Sepsis-3). JAMA.

[2] Rudd KE, Johnson SC, Agesa KM (2020). Global, regional, and national sepsis incidence and mortality, 1990–2017: analysis for the Global Burden of Disease Study. Lancet.

[3] Rose N, Matthaus-Kramer C, Schwarzkopf D, et al. Association between sepsis incidence and regional socioeconomic deprivation and health care capacity in Germany - an ecological study. BMC Public Health. 2021;21(1):1636. 10.1186/s12889-021-11629-4 10.1186/s12889-021-11629-4 PMC8424852 34493250

[4] Fleischmann-Struzek C, Rose N, Freytag A, et al. Epidemiology and Costs of Postsepsis Morbidity, Nursing Care Dependency, and Mortality in Germany, 2013 to 2017. JAMA Netw Open. 2021;4(11):e2134290. 10.1001/jamanetworkopen.2021.34290 10.1001/jamanetworkopen.2021.34290 PMC8590172 34767025

[5] Prescott HC, Angus DC (2018). Enhancing Recovery From Sepsis: A Review. JAMA.

[6] Prescott HC, Langa KM, Iwashyna TJ (2015). Readmission diagnoses after hospitalization for severe sepsis and other acute medical conditions. JAMA.

[7] Fleischmann-Struzek C, Ditscheid B, Storch J, et al. Evaluation of Infection-Related Hospitalizations and Drug Prescriptions Among Sepsis Survivors in Germany. JAMA Netw Open. 2022;5(7):e2220945. 10.1001/jamanetworkopen.2022.20945 10.1001/jamanetworkopen.2022.20945 PMC9270694 35802376

[8] Rhodes A, Phillips G, Beale R (2015). The Surviving Sepsis Campaign bundles and outcome: results from the International Multicentre Prevalence Study on Sepsis (the IMPreSS study). Intensive Care Med.

[9] Latten G, Hensgens K, de Bont E, Muris JWM, Cals JWL, Stassen P. How well are sepsis and a sense of urgency documented throughout the acute care chain in the Netherlands? A prospective, observational study. BMJ Open. 2020;10(7):e036276. 10.1136/bmjopen-2019-036276 10.1136/bmjopen-2019-036276 PMC7371221 32690518

[10] Fleischmann-Struzek C, Rose N, Born S, et al. [White Paper - Improving the care of patients with impairments following sepsis and infections]. Dtsch Med Wochenschr. 2022;147(8):485–491. White Paper - Verbesserung der Versorgungs- und Behandlungsangebote fur Menschen mit Sepsis- und Infektionsfolgen. doi:10.1055/a-1741-3013 10.1055/a-1741-3013 35405753

[11] Winkler D, Rose N, Freytag A (2023). The effects of postacute rehabilitation on mortality, chronic care dependency, health care use, and costs in sepsis survivors. Ann Am Thorac Soc.

[12] Born S, Matthaus-Kramer C, Bichmann A (2023). Sepsis survivors and caregivers perspectives on post-acute rehabilitation and aftercare in the first year after sepsis in Germany. Front Med (Lausanne).

[13] Huang CY, Daniels R, Lembo A (2019). Life after sepsis: an international survey of survivors to understand the post-sepsis syndrome. Int J Qual Health Care.

[14] Reinhart K, Daniels R, Kissoon N, Machado FR, Schachter RD, Finfer S (2017). Recognizing Sepsis as a Global Health Priority - A WHO Resolution. N Engl J Med.

[15] Bone RC, Balk RA, Cerra FB, et al. Definitions for sepsis and organ failure and guidelines for the use of innovative therapies in sepsis. The ACCP/SCCM Consensus Conference Committee. American College of Chest Physicians/Society of Critical Care Medicine. Chest. 1992;101(6):1644–55. 10.1378/chest.101.6.1644 10.1378/chest.101.6.1644 1303622

[16] Kuhlen R, Winklmair C, Schmithausen D, Schick J, Scriba P. The Effects of the COVID-19 Pandemic and Lockdown on Routine Hospital Care for Other Illnesses. Dtsch Arztebl International. 2020 2020;117(27–28):488–9. 10.3238/arztebl.2020.0489 10.3238/arztebl.2020.0488 PMC7575899 33050998

[17] Lamer A, Laurent G, Pelayo S, El Amrani M, Chazard E, Marcilly R (2020). Exploring patient path through sankey diagram: A proof of concept. Stud Health Technol Inform.

[18] Hastings SN, Whitson HE, Sloane R, Landerman LR, Horney C, Johnson KS (2014). Using the past to predict the future: latent class analysis of patterns of health service use of older adults in the emergency department. J Am Geriatr Soc.

[19] Muthen B, Muthen LK (2000). Integrating person-centered and variable-centered analyses: growth mixture modeling with latent trajectory classes. Alcohol Clin Exp Res.

[20] Bakk Z, Kuha J (2021). Relating latent class membership to external variables: An overview. Br J Math Stat Psychol.

[21] Piedmont S, Brammen D, Branse D, Focke K, Kast W, Robra BP. Auf dem Weg zur integrierten Qualitätssicherung im Rettungsdienst. Notfall + Rettungsmedizin. 2018;21(8):682–689. 10.1007/s10049-018-0440-9

[22] Goldhahn L, Swart E, Piedmont S. [Linking Health Claims Data and Records of Emergency Medical Services: Building a Bridge via Patient's Health Insurance Number?]. Gesundheitswesen. 2021;83(S 02):S102-S112. Verknupfung von Abrechnungsdaten gesetzlicher Krankenkassen und Einsatzprotokollen des Rettungsdienstes: Bruckenschlag durch Krankenversichertennummer? doi:10.1055/a-1630-7398 10.1055/a-1630-7398 34852382

[23] Piedmont S, Goldhahn L, Swart E, Somasundaram R, Bauer W. Sepsis im Rettungsdienst: Ihre Relevanz und Früherkennung. . presented at: 16 Jahrestagung Deutsche Gesellschaft Interdisziplinäre Notfall-und Akutmedizin (DGINA) eV; 2021;

[24] Tusgul S, Carron PN, Yersin B, Calandra T, Dami F. Low sensitivity of qSOFA, SIRS criteria and sepsis definition to identify infected patients at risk of complication in the prehospital setting and at the emergency department triage. Scand J Trauma Resusc Emerg Med. 2017;25(1):108. 10.1186/s13049-017-0449-y 10.1186/s13049-017-0449-y PMC5670696 29100549

[25] Casu S, Blau J, Schempf B, Häske D. If you don’t take a temperature, you can’t find a fever. Notfall + Rettungsmedizin. 2019;22(6):509–513. 10.1007/s10049-018-0526-4

[26] Cajöri G, Lindner M, Christ M. Früherkennung von Sepsis − die Perspektive Rettungsdienst. Notfall + Rettungsmedizin. 2019;22(3):189–197. 10.1007/s10049-018-0468-x

[27] Bauer W, Galtung N, von Wunsch-Rolshoven Teruel I, Dickescheid J, Reinhart K, Somasundaram R. Screening auf Sepsis in der Notfallmedizin – qSOFA ist uns nicht genug. Notfall + Rettungsmedizin. 2023; 10.1007/s10049-022-01078-w

[28] Rivaz M, Shokrollahi P, Ebadi A (2019). Online focus group discussions: An attractive approach to data collection for qualitative health research. Nurs Pract Today.

[29] Zwaanswijk M, van Dulmen S. Advantages of asynchronous online focus groups and face-to-face focus groups as perceived by child, adolescent and adult participants: a survey study. BMC Res Notes. 2014;7:756. 10.1186/1756-0500-7-756 10.1186/1756-0500-7-756 PMC4213506 25341440

[30] Gordon AR, Calzo JP, Eiduson R, et al. Asynchronous Online Focus Groups for Health Research: Case Study and Lessons Learned. Int J Qual Methods. 2021;20. 10.1177/1609406921990489 10.1177/1609406921990489 PMC8856649 35185443

[31] Kuckartz U. Qualitative inhaltsanalyse: methoden, praxis, computerunterstützung. Beltz Juventa; 2012.

[32] Mayring P. Qualitative content analysis: A step-by-step guide. Qual Content Anal. 2021:1–100.

[33] Burke JG, O’Campo P, Peak GL, Gielen AC, McDonnell KA, Trochim WM (2005). An introduction to concept mapping as a participatory public health research method. Qual Health Res.

[34] Trochim WM (1989). An introduction to concept mapping for planning and evaluation. Eval Program Plann.

[35] Trochim W, Kane M (2005). Concept mapping: an introduction to structured conceptualization in health care. Int J Qual Health Care.

[36] Pope C, Ziebland S, Mays N (2000). Qualitative research in health care Analysing qualitative data. Bmj.

[37] Sarrazin MS, Rosenthal GE (2012). Finding pure and simple truths with administrative data. JAMA.

[38] Kreis K, Neubauer S, Klora M, Lange A, Zeidler J. Status and perspectives of claims data analyses in Germany—A systematic review. Health Policy. 2016;120(2):213–226. 10.1016/j.healthpol.2016.01.007 10.1016/j.healthpol.2016.01.007 26826756

[39] Schwarzkopf D, Rose N, Fleischmann-Struzek C, et al. Understanding the biases to sepsis surveillance and quality assurance caused by inaccurate coding in administrative health data. Infection. 2023;10.1007/s15010-023-02091-y 10.1007/s15010-023-02091-y PMC10954942 37684496

